# Recrystallization During Annealing of Low-Density Polyethylene Non-Woven Fabric by Melt Electrospinning

**DOI:** 10.3390/polym17152121

**Published:** 2025-07-31

**Authors:** Yueming Ren, Changjin Li, Minqiao Ren, Dali Gao, Yujing Tang, Changjiang Wu, Liqiu Chu, Qi Zhang, Shijun Zhang

**Affiliations:** 1 College of Materials Science and Engineering, Beijing University of Chemical Technology, Beijing 100029, China; renyuem91.bjhy@sinopec.com; 2 Sinopec (Beijing) Research Institute of Chemical Industry Co., Ltd., Beijing 100013, China; lichj.bjhy@sinopec.com (C.L.); renmq.bjhy@sinopec.com (M.R.); gaodl.bjhy@sinopec.com (D.G.); tangyj.bjhy@sinopec.com (Y.T.); chulq.bjhy@sinopec.com (L.C.); zhangqi01.bjhy@sinopec.com (Q.Z.)

**Keywords:** recrystallization, annealing, low-density polyethylene, non-woven fabric

## Abstract

The effect of annealing on the microstructure and tensile properties of low-density polyethylene (LDPE) non-woven fabric produced by melt electrospinning was systematically investigated using DSC, SAXS, SEM, etc. The results showed that, above an annealing temperature of 80 °C, both the main melting point and crystallinity of LDPE decreased compared to the original sample, as did the tensile strength of the non-woven fabric. Additionally, the lamellar distribution became broader at annealing temperatures above 80 °C. The recrystallization mechanism of molten lamellae (disordered chains) in LDPE was elucidated by fitting the data using a Gaussian function. It was found that secondary crystallization, forming thicker lamellae, and spontaneous crystallization, forming thinner lamellae, occurred simultaneously at rates dependent on the annealing temperature. Secondary crystallization dominated at temperatures ≤80 °C, whereas spontaneous crystallization prevailed at temperatures above 80 °C. These findings explain the observed changes in the microstructure and tensile properties of the LDPE non-woven fabric. Furthermore, a physical model describing the microstructural evolution of the LDPE non-woven fabric during annealing was proposed based on the experimental evidence.

## 1. Introduction

Over the past few decades, polyethylene (PE) non-woven fabrics, which feature a web-like structure composed of either oriented or randomly arranged fibers, have played an indispensable role in the medical and healthcare sectors due to their non-toxic nature, skin friendliness, excellent thermal adhesion, and soft handfeel. They are not only ideal for use in surgical gowns, protective clothing, sanitary napkins, and diapers but can also be combined with other materials for applications in automotive interiors, as waterproofing materials, and in various other fields.

Currently, PE non-woven fabrics are manufactured using technologies such as electrospinning, flash spinning, and melt blowing. Among these, electrospinning stands out for its material versatility, low cost, and ease of industrialization [1], making it a widely adopted method in both academia and industry for the efficient large-scale production of nanofibers and microfibers. Electrospinning technology utilizes a high-voltage electrostatic field to charge and elongate the PE solution or melt, forming a cone, known as a Taylor cone, at the tip of the nozzle. Then, when the charge repulsion on the droplet surface exceeds its surface tension, high-speed PE jets are formed.

These jets are stretched by the electric field, and the PE solidifies during their descent (in solution electrospinning, the solvent evaporates prior to solidification), ultimately forming PE nanofibers or microfibers deposited on the collector plate [2]. In recent years, solvent-free melt electrospinning [3], has gained increased attention as a safe, efficient, and environmentally friendly method for producing ultrafine fibers. Yang et al. [4] utilized melt electrospinning to overcome the challenges associated with refining PE fibers and successfully prepared a PE non-woven fabric, although the resulting material still required improvements in mechanical strength.

In general, annealing below the melting temperature of a polymer can reduce structural defects, enhance the crystal structure, and improve the mechanical properties of the material [5,6]. As one of the most common polymers, PE has been the subject of numerous studies investigating the microstructural and macroscopic changes induced by annealing, particularly in polymers with various molecular structures. For example, R. Lam et al. [7] demonstrated the growth of linear PE single crystals from the glassy amorphous state upon annealing just above the glass transition temperature ($T_{g}$). A. S. Maxwell et al. [8] explored the effects of high-pressure annealing on the molecular networks of four grades of high-density linear PE (HDPE), finding that pressure annealing improved the drawability and, consequently, the physical properties of high-molecular-weight materials. S. J. Bai et al. [9] observed that the long period of spherulitic PE increased with the annealing temperature, ranging from 35 to 54 nm. Similarly, S. Tiemprateeb et al. [10] found that raising the annealing temperature led to increases in the melting temperature, crystallinity, modulus, and tensile strength in calcium carbonate–HDPE composites. Moreover, the effects of annealing on other types of PE, including ultra-high-molecular-weight PE (UHMWPE) [11], cross-linked PE (XLPE) [12], and branched PE [13,14], have also been investigated [15,16]. In particular, studies on branched PE have reported that the contribution of crystallinity diminishes at higher annealing temperatures, and a lower melting point is observed for FC-branched PE after annealing. However, to date, no studies have systematically examined the effects of annealing on non-woven fabrics made from melt-electrospun low-density PE (LDPE), which contains branched molecular chains.

Therefore, this study primarily investigates the effects of various annealing temperatures on LDPE non-woven fabric using differential scanning calorimetry (DSC) and small-angle X-ray scattering (SAXS). Gaussian fitting and the electron density correlation function were employed to analyze lamellar structural variations during annealing and to reveal the theoretical mechanisms underlying microstructural changes in LDPE. These findings provide guidance for the post-treatment of LDPE non-woven fabrics in future applications.

## 2. Materials and Methods

### 2.1. Materials

The LDPE used in this study was supplied by Sinopec. Its key properties are listed in Table 1. The melting point ($T_{m}$) and the crystallization temperature ($T_{c}$) were measured using DSC at a heating rate of 10 °C/min under a nitrogen flow of 20 mL/min. The polydispersity index (PDI) and molecular weight were determined using gel permeation chromatography (GPC).

### 2.2. Sample Preparation

The device used for preparing LDPE non-woven fabrics [4] primarily consists of five components: an extruder, a nozzle, a hollow disc, a high-voltage DC power supply, and a roller. The polymer melt generated by the extruder is continuously fed into the nozzle at a constant rate of 10 g/min. A high electrostatic voltage of 50 kV is applied to the hollow disc, while the nozzle is grounded. Under the influence of the electrostatic field, the melt is split into multiple jets, which are subsequently collected by the roller at a speed of 20 m/min to form non-woven fabrics.

The LDPE non-woven fabrics produced by melt electrospinning were annealed in a vacuum oven at 60 °C, 70 °C, 80 °C, 90 °C, and 100 °C for 1 h. After annealing, the samples were sealed in bags for preservation. The samples were named as follows: the LDPE non-woven fabric before annealing is referred to as the PE precursor, while the samples annealed at T °C for 1 h are denoted by PE-ATT, such as PE-AT60.

### 2.3. Characterization

#### 2.3.1. DSC

A PerkinElmer DSC 8000 equipped with a mechanical refrigerator was used to analyze the thermal properties of the PE non-woven fabric. The temperature and heat flow were calibrated using standard materials such as benzoic acid and indium. The dried samples, each weighing approximately 3 mg, were sealed in aluminum pans. To ensure test accuracy, nitrogen gas was purged at a flow rate of 20 mL/min to protect the samples during scanning. All samples were heated from 0 °C to 180 °C at a rate of 10 °C/min to evaluate the melting behavior of the annealed PE.

The crystallinity ($X_{c}$) of PE was calculated using the following equation [17]:

(1) $$X_{c}=\frac{\Delta H_{m}}{\Delta H_{m}^{*}}\times 100\%$$

where $\Delta H_{m}$ denotes the fusion enthalpy of the sample, and $\Delta H_{m}^{*}$ represents the fusion enthalpy of the perfect crystal of PE, equal to 277.1 J/g [18].

#### 2.3.2. SAXS

SAXS patterns were collected using a Bruker AXS Nanostar system. This instrument is equipped with a microfocus copper anode operating at 45 kV/0.65 mA. Montel optics and a VANTEC 2000 2D detector (Bruker Corporation, Wurzbach, German) were positioned 104.7 mm from the samples prepared from the non-woven fabric.

To clearly illustrate the variations in the crystalline phase, amorphous phase, and transition state as a function of the annealing temperature, the one-dimensional (1D) electron density correlation function was calculated, following the methods described in [19,20]:

(2) $$K\left(z\right)=\int_{0}^{\infty }q^{2}I\left(q\right)\cos qzdq$$

where *q* represents the scattering vector, *I*(*q*) is the scattering intensity, and z denotes the distance in real space, normal to lamellae.

#### 2.3.3. Scanning Electron Microscopy (SEM)

To clearly observe the fiber structure of the PE non-woven fabric, the samples were examined using SEM. A Hitachi S4800 microscope (Hitachi, Ltd., Tokyo, Japan) was used for the analysis. The electron beam voltage was set to approximately 5.0 kV. SEM images were captured using a secondary electron detector at magnifications ranging from 50× to 250×.

#### 2.3.4. Tensile Properties

A Linkam TST350 hot stage was used to evaluate the tensile properties of the LDPE non-woven fabric. Dumbbell-shaped specimens were prepared for testing, with a notch length of 6 mm and a width of 4 mm. The stretching rate was set at 100 μm/s.

## 3. Results and Discussion

### 3.1. Morphology of LDPE Fibers in Non-Woven Fabric

**Figure 1:**
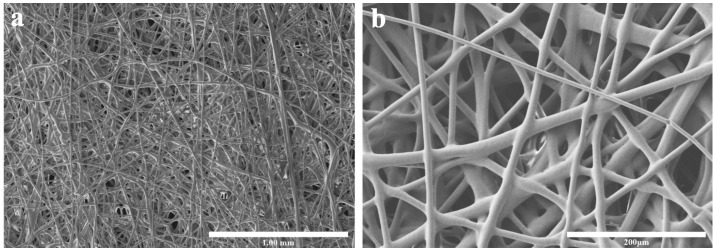
SEM micrograph of precursor PE non-woven fabric for (**a**) magnificated at 50×, (**b**) magnificated at 250×.

**Figure 2:**
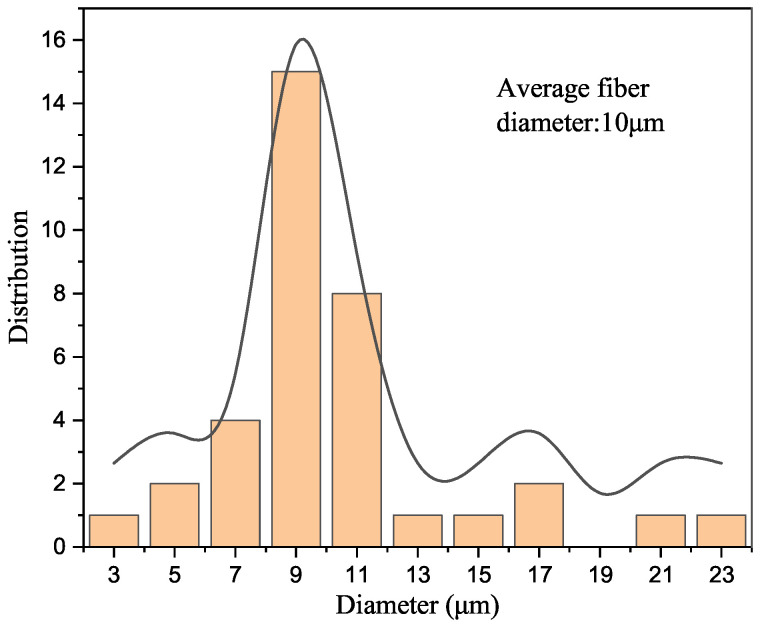
The distribution of the diameters of fibers in the LDPE non-woven fabric.

The SEM micrographs were used to examine the fiber structure of the precursor PE non-woven fabric. Figure 1a,b were obtained at magnifications of 50× and 250×, respectively. As shown in Figure 1, the fibers are randomly entangled, and microfibers with smaller diameters are often distributed near those with larger diameters. Additionally, the fiber diameters in the SEM images were measured using ImageJ (1.54p). Figure 2 presents the fiber diameter distribution, indicating that the average fiber diameter is approximately 10 μm.

### 3.2. Changes in Thermodynamic Behavior of LDPE Non-Woven Fabric During Annealing

**Figure 3:**
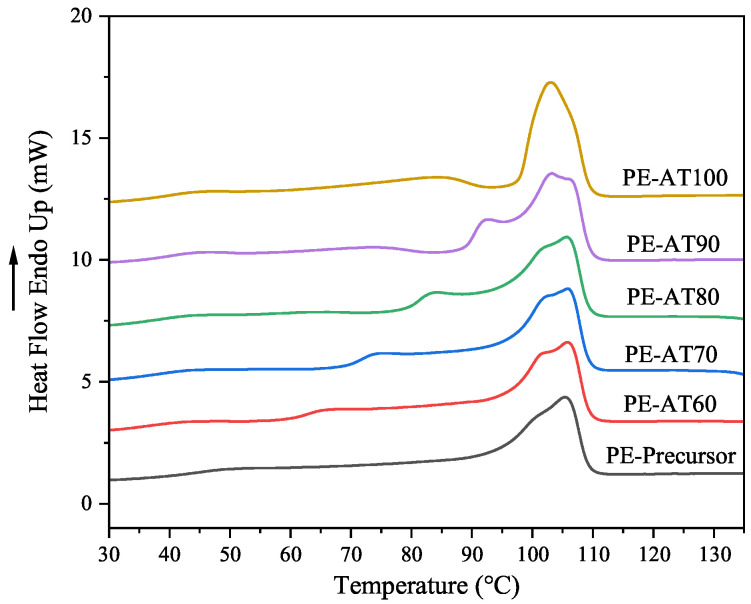
DSC first heating curves of LDPE non-woven fabric at different annealing temperatures for 1 h.

The thermodynamic behavior of the LDPE non-woven fabric was analyzed using DSC, as shown in Figure 3. In the precursor sample, three melting peaks were observed at approximately 50 °C, 100 °C, and 105 °C. The melting peaks around 50 °C indicate the existence of fine crystals. After annealing, a small melting peak appeared slightly above the annealing temperature, which was due to the melting of the recrystallized crystal [21], while the peak near 100 °C became stronger and more pronounced. As the annealing temperature increased, the positions of these melting peaks gradually shifted toward higher temperatures. This shift indicates that the crystalline regions of LDPE become increasingly perfect, until it merges with the main melting peak at an annealing temperature of 100 °C.

Meanwhile, a subtle bulge unexpectedly appeared on the DSC curve below the annealing temperature. As the annealing temperature increased, these bulges became more pronounced, particularly at 100 °C. This may suggest the formation of a more fragmented crystalline structure that was not originally intended during annealing. The melting point and crystallinity of LDPE are summarized in Table 2. As shown, both the crystallinity and main melting point initially increase and then decrease with rising annealing temperatures.

**Figure 4:**
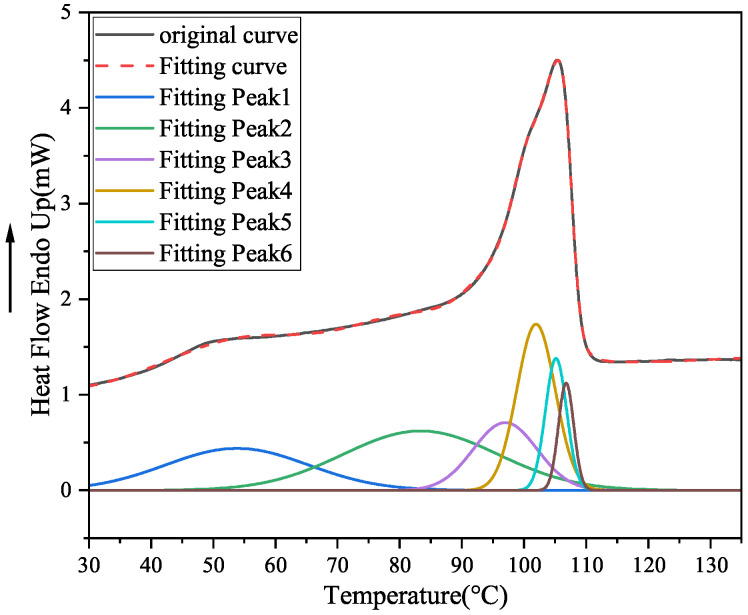
A comparison of the curves before and after the fitting of the DSC melting peak of the PE precursor sample.

To extract hidden information from the DSC curves, analysis software (PeakFit v4.12) was used to decompose the complex thermal signals into independent and more interpretable sub-peaks. During this fitting process, the Gaussian function was applied, and the statistical indicator $R^{2}$ for all samples exceeded 0.999, indicating an excellent fit, as shown in Figure 4.

The fitting results revealed the presence of six sub-peaks in each sample, each corresponding to a lamellar structure with a different thickness. The relationship between the melting point of the polymer and the lamellar thickness of a single polymer crystal can be described by the Gibbs–Thomson equation [22]:

(3) $$T_{m}=T_{m}^{0}-\frac{2\sigma_{e}T_{m}^{0}}{l\Delta h}$$

where $T_{m}^{0}$ represents the melting temperature of an infinitely large crystal (the equilibrium melting temperature), $\sigma_{e}$ is the top and bottom specific surface free energy, l is the lamellar thickness, and ΔH is the bulk heat of fusion per cubic centimeter. According to the equation, the lamellar thickness is positively correlated with the melting point of the polymer.

**Figure 5:**
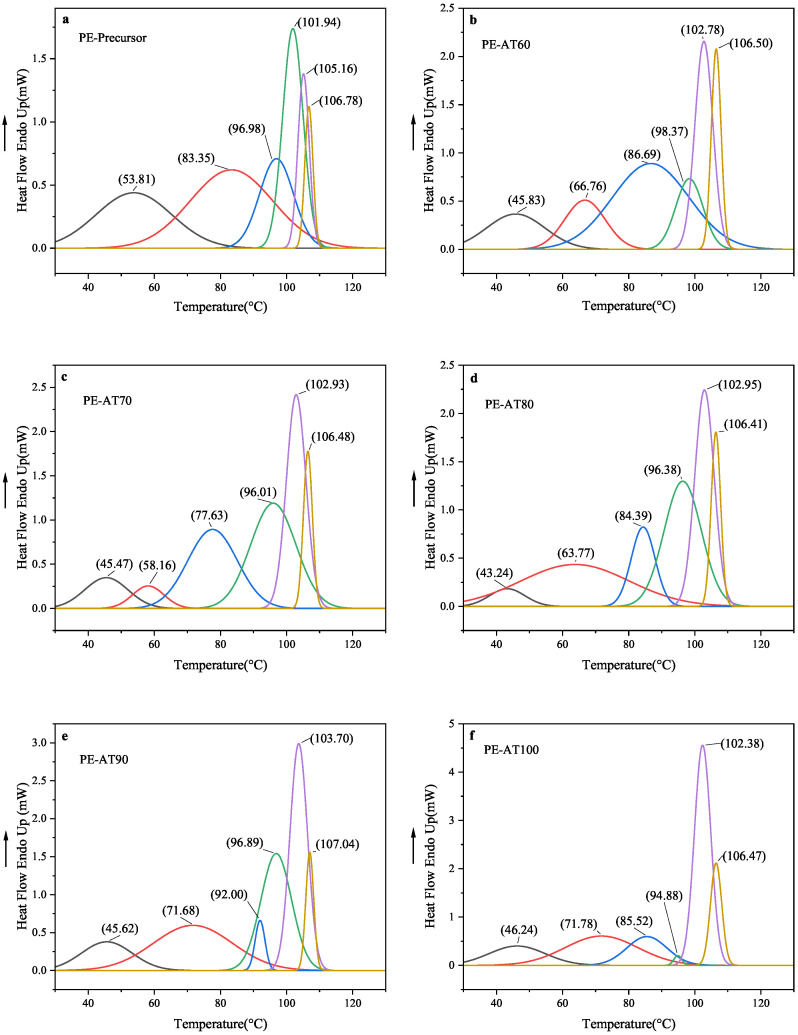
The independent sub-peaks obtained by fitting the DSC data of LDPE at different annealing temperatures for (**a**) unannealed LDPE non-woven fabric; (**b**–**f**) annealed at 60 °C, 70 °C, 80 °C, 90 °C, 100 °C seperately.

Figure 5 and Table 3 show the individual sub-peaks and the peak values obtained by fitting the DSC data of LDPE at different annealing temperatures. A melting peak below the annealing temperature was observed in each case, and the number of these low-temperature peaks increased progressively with rising annealing temperatures, further confirming the previous DSC analysis. When the sample was annealed at 60 °C, a melting peak at 45.83 °C, below the annealing temperature, was observed, indicating the formation of thinner and less stable lamellae compared to the precursor sample. Meanwhile, the melting peaks at 66.76 °C, 86.69 °C, 98.37 °C, 102.78 °C, and 106.50 °C became more prominent, suggesting the enhancement of the original lamellae. For samples annealed at 70 °C, 80 °C, and 90 °C, two melting peaks were present below each respective annealing temperature. The melting peaks of PE-AT80 and PE-AT90 were broader than those of PE-AT70, implying a more uneven distribution of thinner lamellae at 80 °C and 90 °C. Additionally, the high-temperature peak around 106 °C in PE-AT90 was weaker than in PE-AT70 and PE-AT80, suggesting a reduction in the amount of thicker and more perfect lamellae. When the sample was annealed at 100 °C, four melting peaks appeared below the annealing temperature, while only two remained above it. This indicates the significant deterioration of the lamellar structure. These results explain why the crystallinity and main melting point of LDPE initially increase and then decrease with rising annealing temperatures.

### 3.3. Variations in Microstructure, Including Crystalline Phase and Amorphous Phase, of LDPE During Annealing

**Figure 6:**
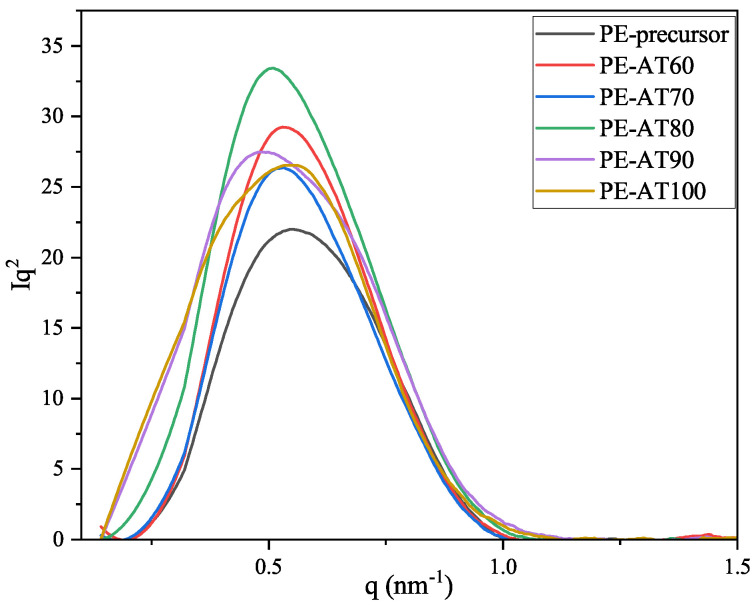
The $Iq^{2}-q$ profile of the LDPE non-woven fabric annealed at different temperatures.

SAXS was employed to investigate the microstructural changes in the LDPE non-woven fabric during annealing. Typically, the one-dimensional light intensity scattering curve must be processed before calculating the long period. For isotropic samples, Lorentz correction is necessary, whereas, for samples with symmetrical structures, it is not required [23]. Since the LDPE non-woven fabric was isotropic, the long period was calculated using SAXS data with Lorentz correction. Figure 6 presents the $Iq^{2}-q$ profile derived from the SAXS data for LDPE samples annealed at 60 °C, 70 °C, 80 °C, 90 °C, and 100 °C. It is observed that the scattering peaks become narrower and gradually shift to the left (toward smaller *q* values) at annealing temperatures of 60 °C, 70 °C, and 80 °C, compared with the precursor sample. This indicates an improvement in the uniformity of the lamellar distribution. However, when the annealing temperature increases to 90 °C or 100 °C, the scattering peaks become broader, suggesting that the lamellar distribution becomes more heterogeneous.

The long period, determined from the *q* value in the $Iq^{2}-q$ profile (Figure 6) using the following equation, represents the sum of the lamellar and amorphous phase thicknesses in the ideal two-phase model, which consists solely of crystalline and amorphous regions.

(4) $$L=\frac{2\pi }{q_{\max}}$$

**Figure 7:**
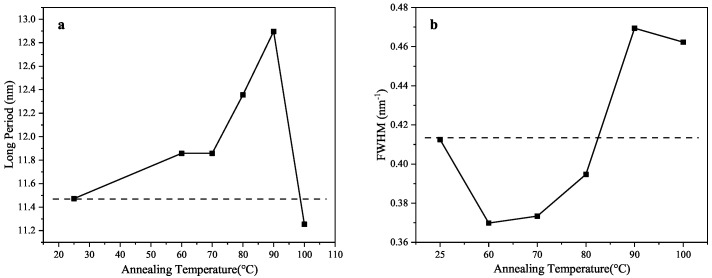
The relationship between (**a**) the long period, (**b**) the full width at half maximum (FWHM), and the annealing temperature (The solid lines represent the variation of parameter, dashed lines are the boundary relative to initial sample).

Here, $q_{max}$ represents the value of *q* on the x-axis corresponding to the peak of the $Iq^{2}$ profile, and L denotes the long period, corrected using the Lorentzian function based on Bragg’s equation. As shown in Figure 7a and Table 4, when the samples were annealed at temperatures below 90 °C, the long period increased gradually with the rising temperature, suggesting an increase in the average thickness of both the lamellar and amorphous phases. However, when annealed at 100 °C, the long period showed a marked decrease, indicating a reduction in the mean thickness of the lamellar and amorphous regions.

The full width at half maximum (FWHM) reflects, to some extent, the uniformity of lamellar distribution: smaller FWHM values suggest a more uniform distribution. As illustrated in Figure 7b and Table 4, the FWHM initially decreased with increasing annealing temperatures and then increased, and it finally showed a slight decline again. Notably, the FWHM values for samples annealed at 90 °C and 100 °C were higher than that of the original sample, while the values for those annealed at 60 °C, 70 °C, and 80 °C were lower. This suggests that annealing below 90 °C enhanced the uniformity of the lamellar distribution.

**Figure 8:**
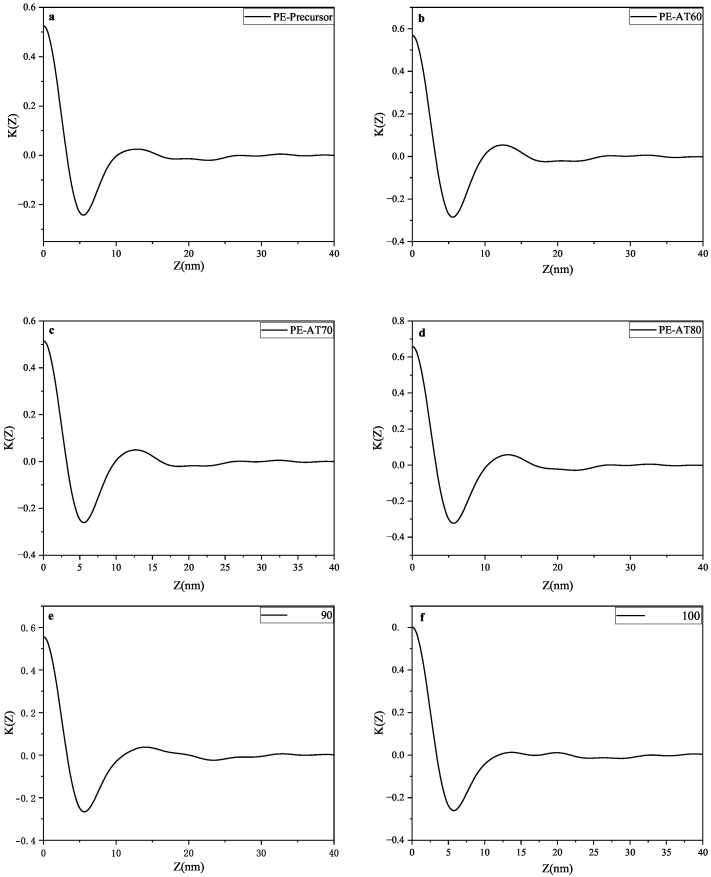
The one-dimensional electron density correlation functions of LDPE samples at different annealing temperatures for (**a**) unannealed LDPE non-woven fabric; (**b**–**f**) annealed at 60 °C, 70°C, 80°C, 90°C, 100°C seperately.

**Figure 9:**
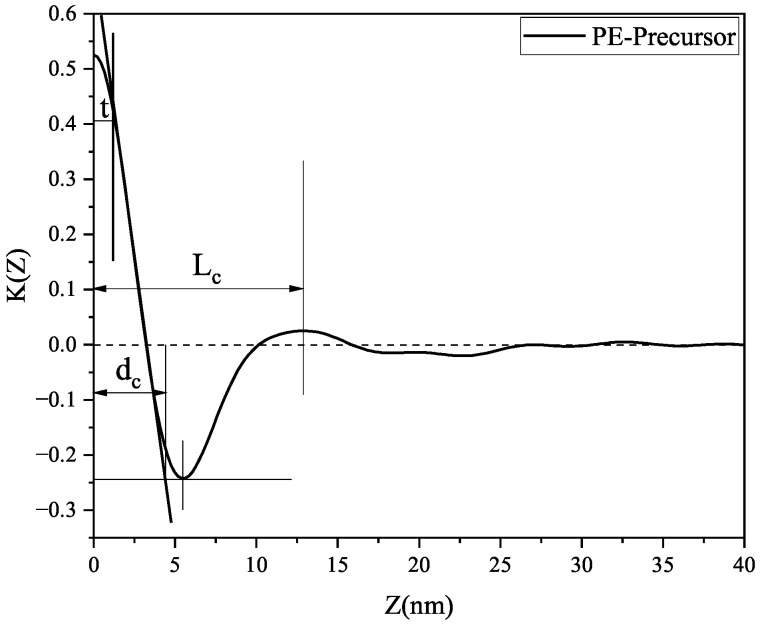
General properties of the electron density correlation function of the PE precursor sample.

**Figure 10:**
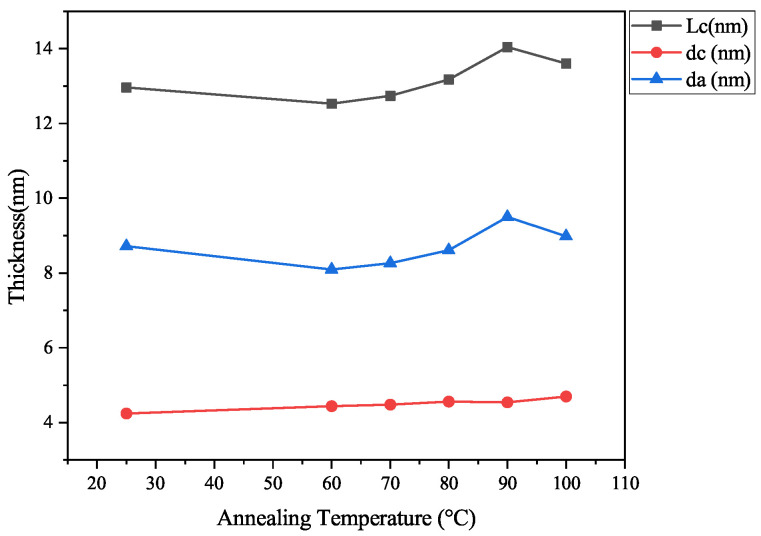
The variation trend in the thickness of $L_{c}$, $d_{c}$, and $d_{a}$ with an increase in the annealing temperature.

The data were analyzed using the one-dimensional electron density correlation function, which reflects the variation in electron density along the lamellar normal. Figure 8 presents the one-dimensional electron density correlation functions of LDPE samples annealed at different temperatures. These experimental functions were evaluated using a method based on the general properties of the correlation function, as illustrated in Figure 9. This figure shows the calculation process for determining $L_{c}$, $d_{c}$, and t, where $L_{c}$ denotes the average distance between adjacent lamellae, $d_{c}$ is the average lamellar thickness (defined as the z-value at which the inclined line intersects the tangent to the first trough), and t represents the average thickness of the interfacial layer. The computed values are summarized in Table 5, and the variation in thickness with increasing annealing temperatures is depicted in Figure 10, where $d_{a}$ represents the mean thickness of the amorphous phase ($d_{a}$ = $L_{c}-d_{c}$). The value of t is negligible compared to the lamellar thickness. As shown in Figure 10, $d_{c}$ remains relatively unchanged with increasing annealing temperatures, whereas $L_{c}$ and $d_{a}$ initially decrease, then increase, and subsequently decrease again. This trend suggests that changes in the average spacing between lamellae are primarily attributed to variations in the amorphous phase thickness. Notably, the electron density correlation function of the PE-AT100 sample (annealed at 100 °C) yielded two distinct $L_{c}$ values, indicating the coexistence of two ordered lamellar structures in the sample.

### 3.4. Discussion of Theoretical Mechanism for Microstructural Changes in LDPE During Annealing

The annealing mechanism of lamellae in polymers is quite complex, involving multiple processes occurring simultaneously. It is generally accepted that the lamellar structure primarily undergoes local melting and recrystallization during annealing [24]. Since the annealing temperature is typically much lower than the thermodynamic equilibrium melting point of the polymer, the crystalline regions can continue to grow during this process, mainly along the chain direction—a phenomenon known as lamellar thickening. Moreover, various non-equilibrium defects within the crystalline regions, such as heterocomponents, branched chains, impurities, and dislocations, are gradually eliminated during annealing. For instance, during the crystallization annealing of PE containing branched chains, the lamellar crystal thickness is limited because the branched chains are excluded from the crystalline regions [25], even under high-pressure conditions [26].

**Figure 11:**
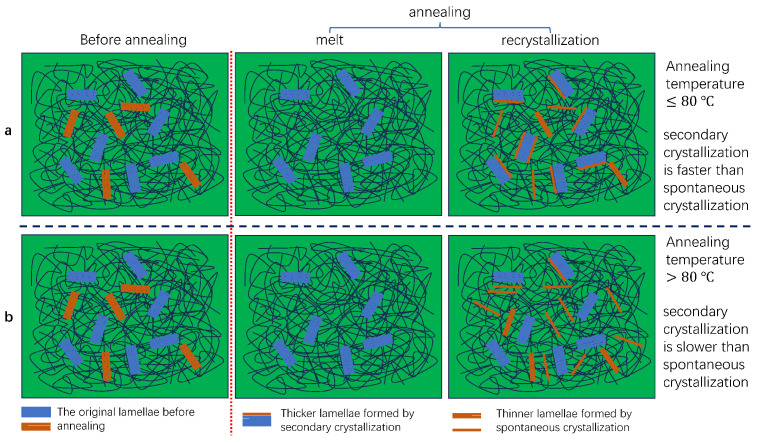
Schematic diagram of the microstructure of LDPE fibers under different annealing temperatures for (**a**) annealing temperature ≤80 °C; (**b**) annealed at above 80 °C.

However, our experimental results indicate that, in LDPE, the extent to which branched chains enter the original lamellae (i.e., contribute to lamellar thickening) is limited. Moreover, the formation of new lamellae, whose melting points are below the annealing temperature, is strongly influenced by the annealing temperature—a phenomenon not reported in previous studies. As illustrated in Figure 11, the changes in the lamellar structure during annealing in LDPE can be classified into two distinct scenarios. First, when the annealing temperature is less than or equal to 80 °C, lamellae with melting points below the annealing temperature undergo melting and subsequent recrystallization. During this process, a large fraction of LDPE molecular chains enters the existing crystalline regions, promoting the growth of the original lamellae through secondary crystallization. The remaining small fraction of molecular chains recrystallizes independently, forming new, thinner lamellae through spontaneous crystallization. These newly formed lamellae have melting points that are lower than the annealing temperature. Additionally, the increase in the amorphous-phase thickness with rising annealing temperatures contributes to an increase in the long period, and the FWHM of the lamellae gradually decreases as the annealing temperature increases, indicating that the lamellar distribution becomes more uniform.

Another scenario arises when the annealing temperature exceeds 80 °C. In this situation, only a small portion of the molecular chains contributes to the thickening of the original lamellae, while the majority recrystallize to form a large number of new lamellae with melting points below the annealing temperature. Meanwhile, the increase in the long period indicates the further thickening of the amorphous phase. The FWHM of the lamellae is higher than that of the precursor sample, suggesting that the lamellar distribution becomes more heterogeneous. Additionally, two distinct long periods, corresponding to two ordered lamellar structures, are observed. One reflects the presence of the thicker original lamellae, while the other indicates the formation and spatial organization of numerous newly formed lamellae.

**Figure 12:**
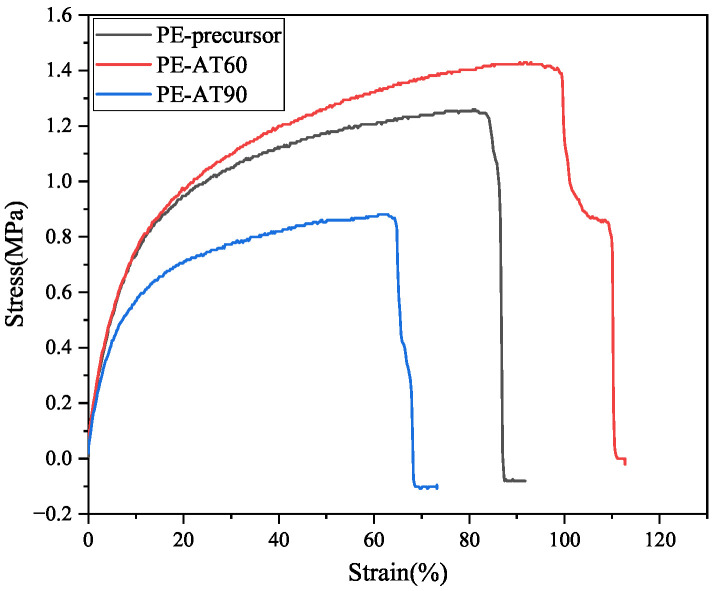
Tensile properties of unannealed LDPE non-woven fabric and that annealed at 60 °C and 90 °C.

The competitive relationship between the newly formed, thinner lamellar structures and the thickening of the original lamellae significantly influences the mechanical properties of LDPE. The presence of these newly formed crystalline structures is generally detrimental to the enhancement of LDPE’s mechanical performance. As shown in Figure 12, when the molten molecular chains of LDPE primarily contributed to the thickening of the original lamellae, as in the PE-AT60 sample, the tensile strength of the non-woven fabric increased. In contrast, when a large portion of the molten molecular chains underwent recrystallization to form new, thinner lamellae compared to the original ones, as in the PE-AT90 sample, the tensile strength of the non-woven fabric decreased.

## 4. Conclusions

For LDPE non-woven fabrics, the recrystallization behavior of molten molecular chains during annealing exhibits two distinct pathways, depending on the annealing temperature. One pathway involves the thickening of existing lamellae, referred to as secondary crystallization. The other involves the formation of new, thinner lamellae compared to the original ones, known as spontaneous crystallization. A competitive relationship exists between these two behaviors. When the annealing temperature is ≤80 °C, the molten molecular chains predominantly contribute to the thickening of the lamellae. However, at temperatures above 80 °C, the molten chains tend to preferentially form new, thinner lamellae. The presence of newly formed, thinner lamellar structures negatively impacts the tensile properties of LDPE non-woven fabrics. These findings have important implications for the post-treatment and application of branched-chain LDPE.

## Figures and Tables

**Table 1:** The main parameters of LDPE.

Trade Name	Supplier	MFR (g/10min) (190 °C, 2.16kg)	Density (g/cm^3^)	$T_{m}$ (°C)	$T_{c}$ (°C)	$M_{w}$	PDI
MG6000	Sinopec	60	0.9186	108.01	90.19	52042	4.99

**Table 2:** The main melting point and crystallinity obtained from the DSC data.

Sample	PE Precursor	PE-AT60	PE-AT70	PE-AT80	PE-AT90	PE-AT100
$T_{m}$ (°C)	105.38	105.78	105.82	105.64	103.28	103.05
$\Delta H_{m}$ (j/g)	108.8	117.65	111.45	111.47	106.58	108.64
Crystallinity (%)	39.26	42.46	40.22	40.23	38.46	39.21

**Table 3:** The peak values of the fitting peaks of samples annealed at different temperatures.

Peak Value	PE Precursor	PE-AT60	PE-AT70	PE-AT80	PE-AT90	PE-AT100
Fitting peak 1 (°C)	53.81	45.83	45.47	43.24	45.62	46.24
Fitting peak 2 (°C)	83.35	66.76	58.16	63.77	71.68	71.78
Fitting peak 3 (°C)	96.98	86.69	77.63	84.39	92.00	85.52
Fitting peak 4 (°C)	101.94	98.37	96.01	96.38	96.89	94.88
Fitting peak 5 (°C)	105.16	102.78	102.93	102.95	103.70	102.38
Fitting peak 6 (°C)	106.78	106.50	106.48	106.41	107.04	106.47

**Table 4:** The long period and FWHM calculated based on the SAXS data.

Sample	PE Precursor	PE-AT60	PE-AT70	PE-AT80	PE-AT90	PE-AT100
Long Period (nm)	11.47	11.86	11.86	12.36	12.90	11.25
FWHM (${\mathrm{nm}}^{-1}$)	0.4125	0.3698	0.3734	0.3947	0.4694	0.4623

**Table 5:** The values of $L_{c}$, $d_{c}$, and $d_{a}$ obtained by the electron density correlation function.

Sample	$L_{c}$ (nm)	$d_{c}$ (nm)	$d_{a}$ (nm)
PE precursor	12.96	4.24	8.72
PE-AT60	12.53	4.44	8.09
PE-AT70	12.74	4.48	8.26
PE-AT80	13.17	4.56	8.61
PE-AT90	14.04	4.54	9.5
PE-AT100	13.60 and 19.87	4.70	8.98 and 15.17

## Data Availability

The original contributions presented in this study are included in the article. Further inquiries can be directed to the corresponding author.
